# Further Insight in the High Selectivity of Pb^2+^ Removal over Cd^2+^ in Natural and Dealuminated Rich-Clinoptilolite

**DOI:** 10.3390/ijms26094154

**Published:** 2025-04-27

**Authors:** Yaneth Stephanie Durán-Avendaño, Norge Cruz Hernández, A. Rabdel Ruiz-Salvador, Mohamed Abatal

**Affiliations:** 1 Facultad de Ingeniería, Universidad Autónoma del Carmen, Ciudad del Carmen C.P. 24155, Campeche, Mexico; 090288@mail.unacar.mx; 2 Departamento de Física Aplicada I, Escuela Politécnica Superior, Universidad de Sevilla, E-41011 Seville, Spain; 3 Departamento de Sistemas Físicos, Químicos y Naturales, Universidad Pablo de Olavide, Carretera de Utrera km. 1, E-41013 Seville, Spain; rruisal@upo.es; 4 Centro de Nanociencia y Tecnologías Sostenibles (CNATS), Universidad Pablo de Olavide, Carretera de Utrera km. 1, E-41013 Seville, Spain

**Keywords:** clinoptilolite, dealumination, heavy metals, ion-exchange, DFT, simulated annealing

## Abstract

This research aims to understand the experimental results on the high selectivity of Pb^2+^ removal over Cd^2+^ in natural and dealuminated rich-clinoptilolite. For this purpose, we have considered the results of experimental and Density Functional Theory (DFT)-based simulated annealing (SA) on sorption of Pb^2+^ and Cd^2+^ from aqueous solution. The dealumination process of natural clinoptilolite (Nat-CLI) was done by H_2_SO_4_ solutions at different concentrations (0.1–1.0 M). The results show that the maximum sorption capacity (q_,max_) of Pb^2+^ and Cd^2+^ varied from 224.554 × 10^−3^ to 53.827 × 10^−3^ meq/g, and between 39.044 × 10^−3^ to 20.529 × 10^−3^ meq/g, respectively, when the values of Si/Al ratio change from 4.36 to 9.50. From a theoretical point of view, the global minimum energies of natural and dealuminated clinoptilolites before and after sorption of Pb^2+^ and Cd^2+^ were calculated by an SA method, where heating-cooling cycles were modeled by ab initio Molecular Dynamics followed by energy minimization. The theoretical results confirmed that for all Si/Al ratios, the sorption of Pb^2+^ and Cd^2+^ takes place, and for dealuminated systems, the exchange energy outcomes are more favorable for the Pb^2+^ cations. Since such energy differences are very small, it is not explained from a thermodynamic point of view. On the other hand, it could be understood from a kinetic perspective. In this way, we set that the atomic structural properties of the zeolite modify the first hydration coordination sphere of metal cations.

## 1. Introduction

Lead (Pb) and cadmium (Cd) are classified by the U.S. Environmental Protection Agency (U.S.EPA, Washington, DC, USA), World Health Organization (WHO, Geneva, Switzerland), and European Union (EU, Brussels, Belgium) as priority inorganic pollutants due to its toxicity and [1] negative effects on humans and ecosystems even at low concentrations. The range of permissible limits of Pb and Cd in drinking water established by the US-EPA, WHO, and EU was 0.01–0.015 mg/L and 0.003–0.010 mg/L, respectively [1]. With the aim of reducing toxicity levels in the water, numerous studies have reported the performance of natural zeolites in the removal of hardness [2] and heavy metals [3] from wastewater as well as in petroleum refining [4], agriculture, and construction [5]. In this way, Clinoptilolite (CLI), which is one of the most widely available natural zeolites, has been commonly used for the removal of inorganic and organic contaminants from water due to its particular physical and chemical properties [6], such as large specific surface, flexibility, well-defined micropore sizes and high chemical and physical stability (e.g., thermal and mechanical) [7].

Zeolites are crystalline alumino-silicates with a framework comprised of tetrahedral building units TO_4_ (T = Si or Al) tetrahedra, connected with corner-sharing oxygen atoms forming complex 3-dimensional structures. It forms cages (0.2–1.2 nm) [8], which are connected by channels containing H_2_O molecules and exchangeable cations [9]. The partial substitution of Si^4+^ by Al^3+^ produces a negative charge in the lattice zeolite which is compensated by the exchangeable alkali and alkaline earth cations (Na^+^, K^+^, Ca^2+^, Sr^2+^ or Ba^2+^) [10] or typically by H^+^ cations for acidic zeolites [11]. These cations can be exchanged by other cations in the contact solution [12]. CLI (International Zeolite Association, IZA code HEU) is isostructural to heulandite and exhibits a Si/Al ratio between 4.5 and 5.5. CLI has a monoclinic structure belonging to the C2/m space group with a unit cell dimension of a =17.66 Å, b = 17.96 Å, c = 7.40 Å, and β = 116°47′ [13]. Its structure consists of two-channel systems running parallel to each other and to the c axis: channels of 8 and 10 tetrahedral cavities with effective diameter of the access windows into the channels and cavities of about 4.4 × 3 Å and 7.9 × 3.5 Å respectively, where can host water molecules and exchangeable cations as Na^+^, K^+^, Ca^2+^, Mg^2+^ [14,15].

Various investigations have studied the capture of heavy metals, such as Pb^2+^ and Cd^2+^, from aqueous solutions on natural and modified zeolites [16,17,18,19,20,21,22,23,24,25,26,27,28,29,30,31]. It has been reported that in a single or in a multi-ions system, the affinity of the sorption process is higher for Pb^2+^ than for Cd^2+^. Langmuir and Freundlich isotherm models have been used to investigate the sorption mechanism. Various investigations reported a well fit of the Freundlich model compared to the Langmuir model. Based on the Freundlich distribution coefficient, (K_F_ (Pb^2+^ = 5.11 L/g) and K_F_ (Cd^2+^ = 0.31 L/g). The affinity was higher for Pb^2+^ than Cd^2+^ [16]. The order of selectivity of zeolites on the removal of heavy metals has been attributed to various factors such as ionic radius, dissociation constant (Pb^2+^ > Cd^2+^) [32], as well as, the electronegativity of cations (electronegativity of Pb^2+^ and Cd^2+^ are 2.33 and 1.69, respectively) [33]. It is eye-catching that these properties concern the cations only and no mention of the zeolite properties has been regarded. However, it has been shown that metal selectivity can be different at varying zeolites or, in general, the ion exchange type or the modifications made on them [34,35,36]. The relevant role of the zeolite structural features on the ion exchange selectivity has been highlighted by Caputo and Pepe [37,38].

Among chemical treatments on zeolites, those changing the chemical composition of the zeolite framework and particularly the Si/Al ratio have a larger impact on ion-exchange properties as they are directly linked to the cation exchange capacity (CEC) of the zeolite. In order to evaluate CEC, we can calculate the total amount in milliequivalents of available cations for exchange per unit mass [39]. Acid treatments produce dealumination and thus reduce the CEC, while potentially changing the local energy landscape in the zeolite might lead to changes in the ion-exchange selectivity. Therefore, reducing the CEC dealumination could be an appropriate modification in cases where the interest was the selectivity. The major crystal phase of CLI does not change after mild acid treatments, [29,40,41,42], however detrimental crystallinity has been observed, and even they can cause a small degree of dissolution of crystalline components by increasing the acid concentration [40,43,44], In addition, the modification of natural zeolite by acid washing may remove pore-blocking impurities, protonate and dealumination of the structure, creating more available active sites for sorption [45].

DFT-based theoretical studies are gaining interest worldwide to understand metal removal with zeolites. Sellaoui et al. [46] studied the uses of a synthesized faujasite-type zeolite Y as an adsorbent for the removal of silver (Ag^+^), copper (Cu^2+^), and cobalt (Co^2+^) from a theoretical point of view, as well as, experimentally. In order to understand and characterize the ion exchanges involved in the removal of all metals, two theoretical approaches based on statistical physics modeling and DFT were applied. Both statistical and DFT approaches agreed that the exchange of Ag^+^ in zeolite Y was easier than Cu^2+^ and Co^2+^.

Awuah et al. [47] studied the sorption of arsenic acid (AsO(OH)_3_) and arsenous acid (As(OH)_3_) on the anhydrous and hydrated Al(III)-modified natural zeolite clinoptilolites using DFT calculations. The calculated sorption energies indicated the potential of these materials for arsenic immobilization. However, the sorption was less favorable for hydrated zeolite with four water molecules, although is an exothermic process for some sites. In addition, when increases the Si/Al ratio in the Al(III)-modified clinoptilolite, the interaction of the arsenic complexes decreases.

The exposed background stimulate questioning whether acid-treated partially dealuminated Clinoptilolite can be used for modifying the ion-exchange selectivity of metal cations of environmental and industrial relevance, such as Pb^2+^ and Cd^2+^. Another emerging question concerns the basis of selectivity, i.e., whether it is dominated by thermodynamic or kinetic factors. In this work, we exploit our previously developed simulated annealing-based DFT exploration to find the lowest energy configurations and thus provide the energy of the metal–exchange reactions in zeolites, by obtaining the global minimum for each system included in the reaction. At first glance, this allows a thermodynamics analysis, and to compare with experimental results conducted for this purpose.

## 2. Results and Discussions

### 2.1. Experimental Results

#### 2.1.1. Characterization

The determination of the zero-point charge of the adsorbent is useful because the adsorption of metal ions depends on the solution pH. The results indicate that the pH_PZC_ of Nat-CLI, CLI-0.1 M, CLI-0.2, CLI-0.5 M, and CLI-1.0 M were 6.20, 3.08, 2.80, 2.65, and 2.54, respectively. The zeolite surface is positively charged when the solution pH is lower than pH_PZC_, favoring anion adsorption, while at pH higher than pH_PZC_, the adsorbent surface is negatively charged, favoring cation adsorption. The decrease in pHpzc indicates that acid treatment of natural zeolite modifies its surface properties. In addition, increasing the sulfuric acid concentration (from 0.1 to 1.0 M) results in more positively charged surfaces.

**Figure 1:**
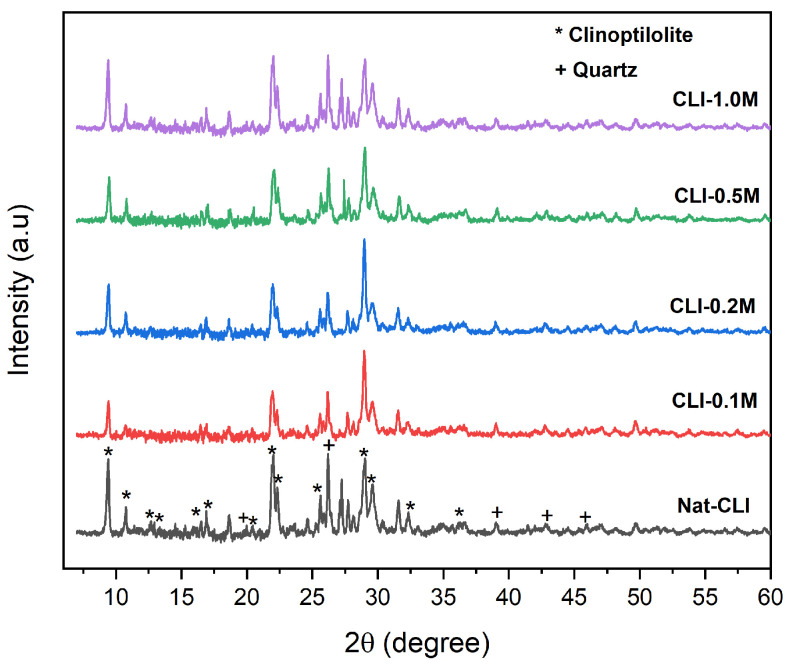
X-ray Diffractograms of the natural and dealuminated clinoptilolites.

Figure 1 shows the XRD results of natural and acidified-clinoptilolites. The XRD patterns indicated that the characteristic peaks of the natural and dealuminated zeolites correspond to clinoptilolite and are in agreement with the JCPDS record (No. 25-1349). After the dealumination, no new peaks appear indicating that any new phases are present. In addition, the crystallinity of the acid-treated samples was found to be slightly lower than that of the Nat-CLI samples. This result can be attributed to the partial dealumination of the clinoptilolite structure [42].

**Figure 2:**
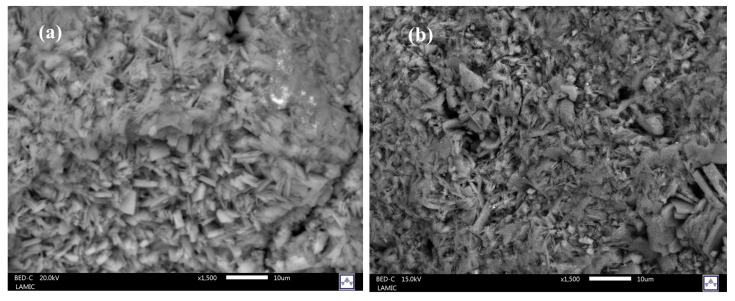

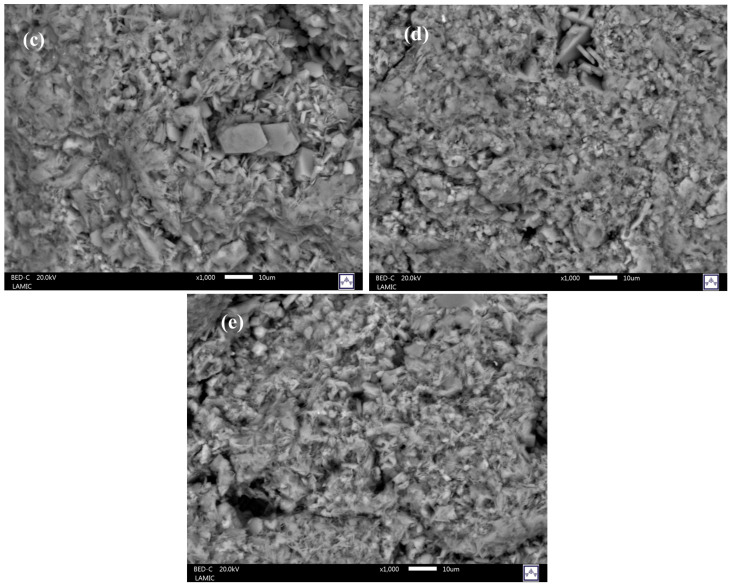
SEM micrographs of (**a**) Nat-CLI, (**b**) CLI-0.1 M, (**c**) CLI-0.2 M, (**d**) CLI-0.5 M, and (**e**) ClI-1.0 M samples.

Scanning electron microscopy (SEM) images of untreated and acid-treated zeolite exhibited tabular, lamellar, and coffin-shaped structures that are characteristic of clinoptilolite (Figure 2) [48]. This result is in agreement with previous research [49].

The average chemical composition of the untreated and acid-treated zeolites was determined by various spot analyses on the surface of the samples using energy-dispersive X-ray spectroscopy (EDS). As shown in Table 1, it can be seen that, after the initial acid treatment (0.1 M), sodium is completely removed. Whereas, for other cations, a gradual decrease in their weight percent was observed as acid concentration increased. For instance, it was found that the weight percent of calcium and potassium decreased from 1.65 and 1.17, respectively, for the natural zeolite to 0.22 and 0.46, respectively, for the acid-treated sample with 1.0 M H_2_SO_4_. In the case of magnesium, the complete elimination of this cation was observed following acid treatment with 0.2 M H_2_SO_4_. Iron is an element frequently found as an impurity in natural zeolites, often in the form of oxides or within the framework at defective sites. EDS analysis showed that the iron content decreased from 0.37 to 0.07, demonstrating the efficacity of the acid treatment in purifying the material [50].

The significant decrease in the atomic percentage of exchangeable cations with acid treatment can be attributed to the partial decationization associated with the dealumination (as explained below) and the ion exchange with the H_3_O^+^ cations. The elimination of these cations from the external surface or within the micropores of natural zeolite is compensated by the incorporation of protons (H^+^) into the framework, increasing Brønsted acidity, which is essential in many catalytic and adsorption processes [50]. 

**Figure 3:**
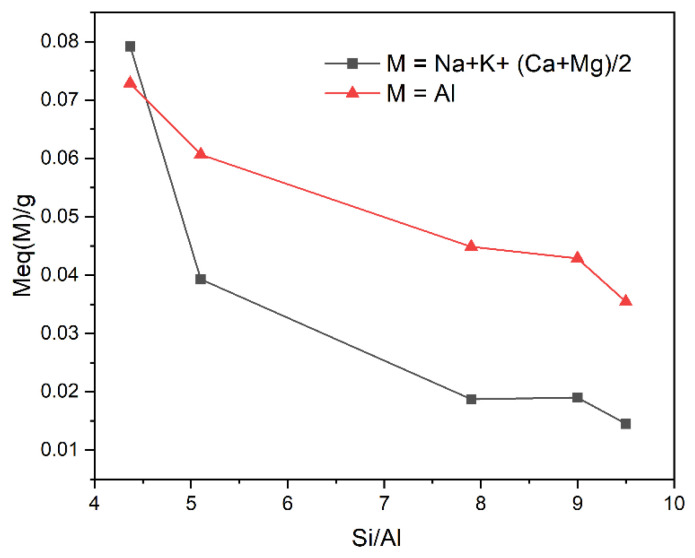
Variation of Si/Al ratio vs. concentration, in meq/g of Al and a total of exchanged cations (Na + K + (Mg + Ca)/2).

Figure 3 shows the variation of Al content, as well as, the sum of monovalent and divalent exchangeable cations (Na + K + (Ca + Mg)/2) versus Si/Al ratio. The Si/Al ratio of the natural zeolite was found to be 4.5, suggesting a framework composition with 29.5 and 6.5 Si and Al, respectively, based on 36 tetrahedral sites in a clinoptilolite cell. It was found that when the sulfuric acid concentration increases from 0.1 M to 1.0 M, the Si/Al ratio increases from 4.5 to 9.5. As the Si/Al ratio increases, the total concentration of exchangeable cations decreases, and the Al content decreases from 0.079 to 0.014 meq/g per cell. This suggests that, in the case of acid-treated zeolites, the decrease of the native extra-framework cations (Na^+^, K^+^, Mg^2+^, and Ca^2+^) is compensated by H^+^ through ion–exchange [51]. This result is confirmed by the decrease in pH_PZC_ of the natural zeolite after acid treatment.

#### 2.1.2. Kinetic Study

**Figure 4:**
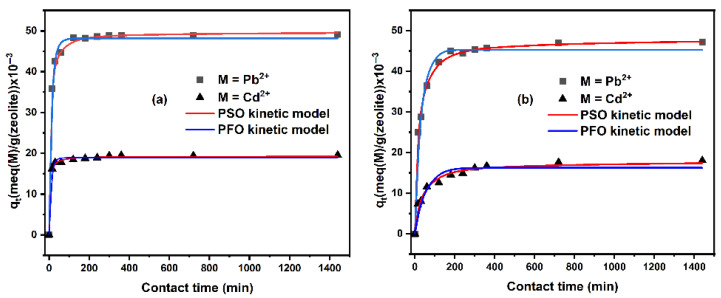

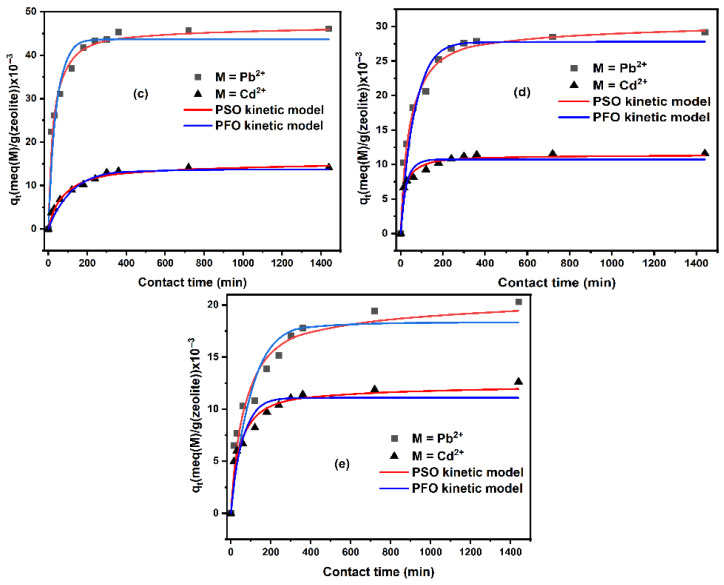
Kinetic modeling of Pb^2+^ and Cd^2+^ according to pseudo-first order and pseudo-second order nonlinear models by (**a**) Nat-CLI, (**b**) CLI-0.1 M, (**c**) CLI-0.2 M, (**d**) CLI-0.5 M, and (**e**) CLI-1 M.

Figure 4 shows the adsorption kinetics data of Pb^2+^ and Cd^2+^ by untreated and dealuminated zeolites and their modeling using the nonlinear PFO and PSO equations. It can be observed that for all adsorbent materials, 53% to 97% of Pb^2+^, and 63% to 94% of Cd^2+^ were removed during the first 120 min. This percentage increases as the Si/Al ratio decreases; after this time, the adsorption capacity gradually reaches equilibrium. This behavior is due to the fact that during the initial adsorption phase, metal ions are more present in the solution and to the availability of exchange ions [52].

The kinetic parameters were calculated using the nonlinear PFO and PSO kinetic models. The results are shown in Table 2. For Cd^2+^, the ranges of correlation coefficients (R^2^) obtained from the PFO and PSO models were R^2^ = 0.662 to 0.938, and 0.912 to 0.981, respectively. Furthermore, it can be observed that the calculated equilibrium sorption capacities (q_e,cal_) by PSO are closer to the experimental equilibrium adsorption capacities (q_e,exp_) compared to the q_e,cal_ obtained from PFO. Moreover, the results obtained from the error functions show lower values for PSO (Table S1 in the Supplementary Material). These results confirm the best PSO model to describe the adsorption kinetics of Pb^2+^. Similar behavior was observed for Cd^2+^ kinetics. As shown in Table 2, the q_e,cal_ for natural and dealuminated zeolites obtained from the PSO model are similar to the q_e,exp_. Also, the values of the error functions are lower for the PSO kinetic model (Table S2 in the Supplementary Material). Therefore, the pseudo-second-order model was found to be the best to describe the sorption kinetics of Pb^2+^ and Cd^2+^. Furthermore, it can be observed from Table 1, that for both metal ions, the pseudo-second-order rate constant (k_2_) and the calculated sorption capacity decrease with increasing Si/Al ratio, this result can be explained by the competition between the metal ions in the solutions and the H^+^ ions which increases with increasing Si/Al.

#### 2.1.3. Isotherm Study

**Figure 5:**
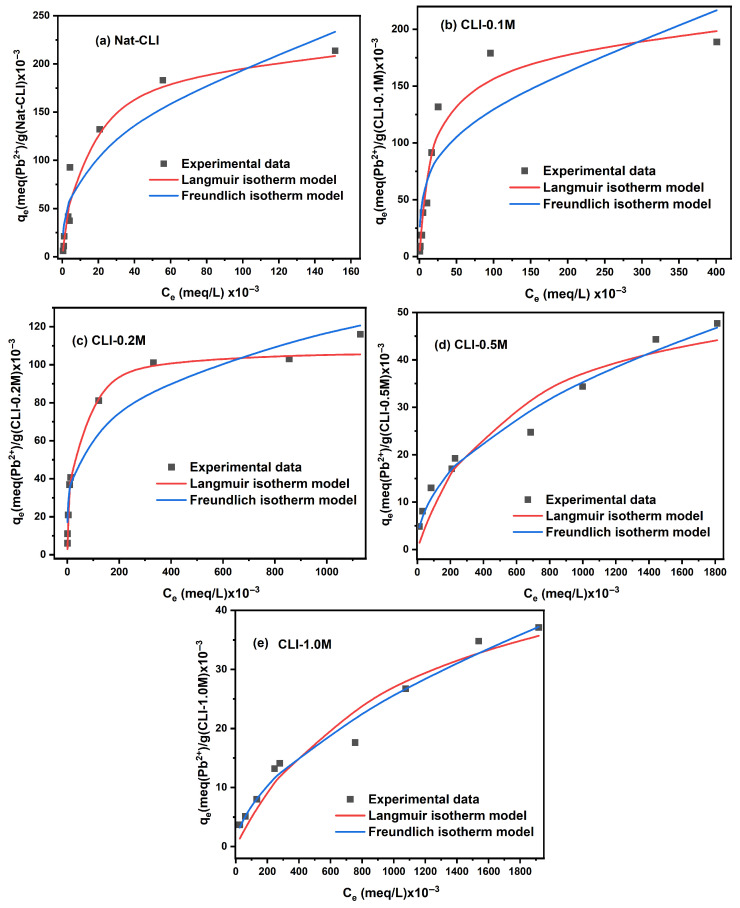
Fitting data for Langmuir and Freundlich nonlinear isotherms for Pb^2+^ adsorption onto (**a**) Nat-CLI, (**b**) CLI-0.1 M, (**c**) CLI-0.2 M, (**d**) CLI-0.5 M, and (**e**) CLI-1.0 M.

**Figure 6:**
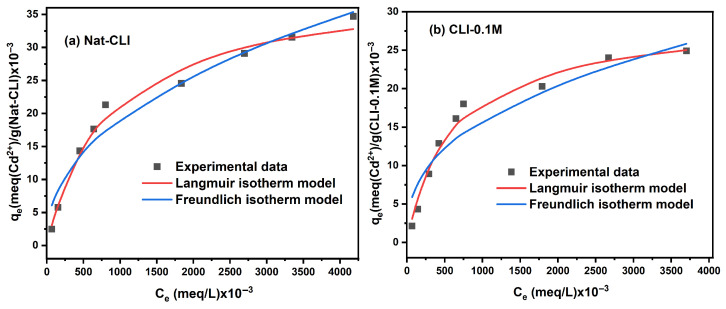

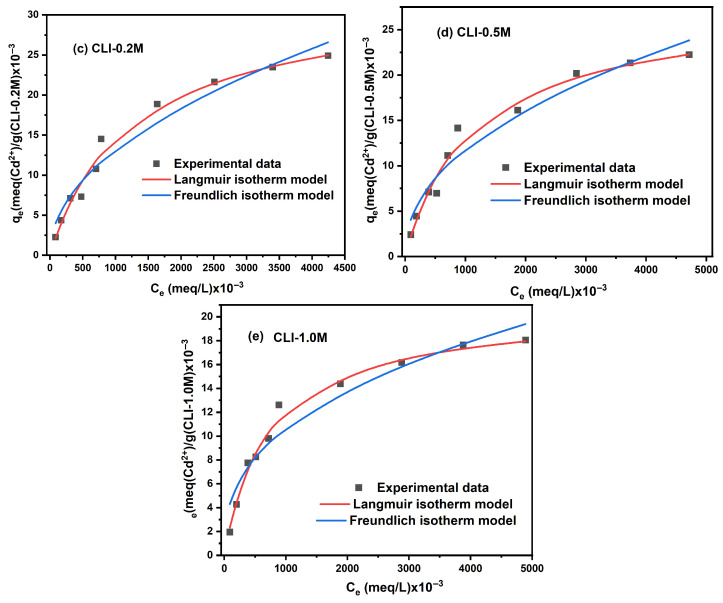
Fitting data for Langmuir and Freundlich nonlinear isotherms for Cd^2+^ adsorption onto (**a**) Nat-CLI, (**b**) CLI-0.1 M, (**c**) CLI-0.2 M, (**d**) CLI-0.5 M, and (**e**) CLI-1.0 M.

The isotherm study was performed to describe the interaction between metal ions and adsorbent surface, as well as to determine the maximum adsorption capacities of untreated and dealuminated zeolites. Figure 5 and Figure 6 show respectively the adsorption isotherms for Pb^2+^ and Cd^2+^ fitted to the Langmuir and Freundlich models. It can be observed that the adsorption capacities for both metal ions increase with increasing metal concentration in the aqueous solution.

Table 3 presents the parameters of the Langmuir and Freundlich isotherm models using the experimental data. In the case of Pb^2+^, it can be seen that, for the samples with 4.36 ≤ Si/Al ≤ 7.9, the Langmuir isotherm was a better fit than the Freundlich isotherm, since the higher values of correlation coefficients (R^2^ = 0.964–0.979) and lower error functions (Table S3 in SI). For the samples with higher Si/Al ratios (Si/Al = 9.04 and 9.50), the Freundlich isotherm was more suitable to describe the sorption equilibrium data.

The Langmuir model assumes a homogeneous surface of the biosorbent, an identical energy distribution on the surface of the adsorbents, and the formation of monolayers [53]. The K_L_ Langmuir constant indicates the binding site affinity and adsorption energy. A low value of K_L_ is related to a low binding site affinity [54]. As shown in Table 3, the values of K_L_ for Pb^2+^ decrease from 8.429 × 10^−2^ to 5.127 × 10^−2^ L/meq whereas the Si/Al ratio increases from 4.37 to 7.9. Therefore, it can be assumed that as the Si/Al ratio increases, the interaction is weaker between metal ions and adsorbents.

The Freundlich model assumes heterogeneous surfaces, reversible adsorption, and multilayer formation. The adsorption process is favorable when the parameter *n* is between 1 and 10 [54], n >1 indicates a physical process, whereas for n < 1, the adsorption is carried out by a chemical process. In our study, the values of n were 2.150 and 1.766 for Si/Al = 9.04 and 9.50, respectively, indicating that the adsorption of Pb^2+^ by CLI-0.5 M and CLI-1.0 M are favorable and regulated by a physical process.

As shown in Table 3, the Langmuir isotherm best fits the adsorption equilibrium data for Cd^2+^. The lower values of the error functions values corroborate the best fit of the Langmuir isotherm model (Table S4 in SI). It can be seen that the maximum adsorption capacity and K_L_ values decrease with increasing Si/Al ratio.

**Figure 7:**
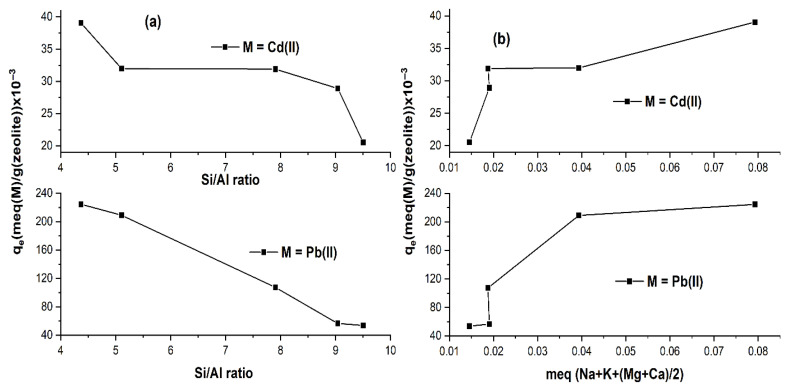
Sorption capacity of Pb^2+^ and Cd^2+^ vs. (**a**) Si/Al, and (**b**) meq of (Na + K + (Ca + Mg)/2).

The decrease of the total amount of exchangeable cations (linked to CEC) with dealumination, shown above in Figure 3, suggests that the amounts of removed Pb^2+^ and Cd^2+^ are likely to decrease with the increase in Si/Al ratio. Figure 7a indeed shows this behavior, e.g., the continuous decrease of metal removal from 224.554 × 10^−3^ to 53.827 × 10^−3^ meq/g in the case of Pb^2+^, while for Cd^2+^ it goes from 39.044 × 10^−3^ to 20.529 × 10^−3^ meq/g. The profiles of the curves representing the metal removal are similar for Pb and Cd, as shown in Figure 7b. However, it is important to note that dealumination does not revert the selectivity towards Cd, being Pb preferably removed in all cases. However, we have noted that both Pb and Cd can be fully exchanged in HEU zeolites at higher temperatures and several days of reaction [55,56], which contrasts with our current result and thus it could indicate that the experimentally observed selectivity could be driven by kinetic factors rather than thermodynamic.

#### 2.1.4. Comparation of Adsorption Capacity of Pb^2+^ and Cd^2+^ with Other Zeolitic Materials

The adsorption of Pb^2+^ using zeolites has been extensively studied as an efficient and cost-effective strategy for treating heavy metal-contaminated water. Despite their natural abundance and low cost, the adsorption performance of unmodified zeolites can be limited by factors such as relatively low surface area, reduced accessibility of internal sites, and competition from coexisting ions in complex matrices. Table 4 compares the maximum adsorption capacities of Pb^2+^ in the present work and by various reporting studies using natural and chemically modified zeolites under different experimental conditions. NaOH-treated zeolite exhibited the highest capacity, reaching 0.929 meq/g under the optimized conditions of pH 6, 27 °C, and 2 h of contact time [57]. This substantial enhancement is ascribed to the impact of alkaline desilication, a process that enhances mesoporosity and improves accessibility to active adsorption sites. Another notable case is the Fe (III)-modified clinoptilolite, which achieved 0.642 meq/g at pH 4.24 and 60 °C [58]. The enhanced performance of this material can be attributed to the formation of Fe-oxihydroxide groups that enhance surface complexation with Pb(II). Similarly, NaCl-pretreated clinoptilolite exhibited its capacity, reaching 0.591 meq/g, in comparison to its natural form compared to its natural form (0.391 meq/g), highlighting how ion exchange with Na^+^ improves site availability [59]. Conversely, certain natural zeolites exhibited considerably diminished adsorption capacities. For instance, natural scolecite demonstrated a capacity of 0.028 meq/g [60], a figure attributable to its compact structure and comparatively diminished cation exchange capacity. A comparable trend was observed in unmodified Iranian clinoptilolite, which exhibited a capacity of 0.035 meq/g [61], a figure that is significantly lower than that of its modified forms.

Table 5 presents a summary of the adsorption capacities and experimental conditions for various natural and modified zeolites used in cadmium removal studies. Among the natural zeolites, Brazilian scolecite showed the highest adsorption capacity reaching 0.483 meq/g, under optimal conditions of pH 6.0, 25 °C, and a high dosage of 16 g/L [65]. This remarkably high value may be related to the increased surface area and ion exchange capacity of this specific material, which also reflects the benefit of using highly porous structures at optimal pH for Cd^2+^ uptake. In contrast, scolecite from a different origin reported a much lower value (0.002 meq/g) under otherwise similar conditions [60], indicating the critical role of mineral origin and structural variability in adsorption performance. Unmodified zeolites studied by Alvarez-Ayuso et al. (2003) [66] and Nguyen et al. (2015) [67] demonstrated low to moderate adsorption capacities (0.041 meq/g and 0.06 meq/g, respectively), likely limited by the competition between Na^+^ and Cd^2+^ at exchange sites and possible saturation effects at higher doses or concentrations.

In contrast, chemically modified zeolites generally showed improved Cd^2+^ uptake due to surface functionalization or structural activation. For instance, NaCl-modified zeolite exhibited a significant enhancement (0.122 meq/g), benefiting from increased exchangeable Na^+^ ions that facilitate Cd^2+^ substitution [68]. Similarly, surfactant-modified zeolite (SMZ), prepared with HDTMA, reached a capacity of 0.090 meq/g under identical conditions [68], showing only a slight decrease due to partial blockage of cation-exchange sites by surfactant molecules, although SMZ may retain the ability to adsorb organic and anionic pollutants simultaneously.

The comparative analysis of Pb (II) and Cd (II) adsorption onto natural and chemically modified zeolites reveals the significant influence of material characteristics and operational parameters on adsorption performance.

In the following section, we present a theoretical study based on DFT calculation to explain the selectivity and adsorption mechanism of Pb and Cd ions by clinoptilolite with different Si/Al ratios.

### 2.2. Theoretical Study

#### 2.2.1. DFT Modeling of Natural and Dealuminated Clinoptilolite

**Figure 8:**
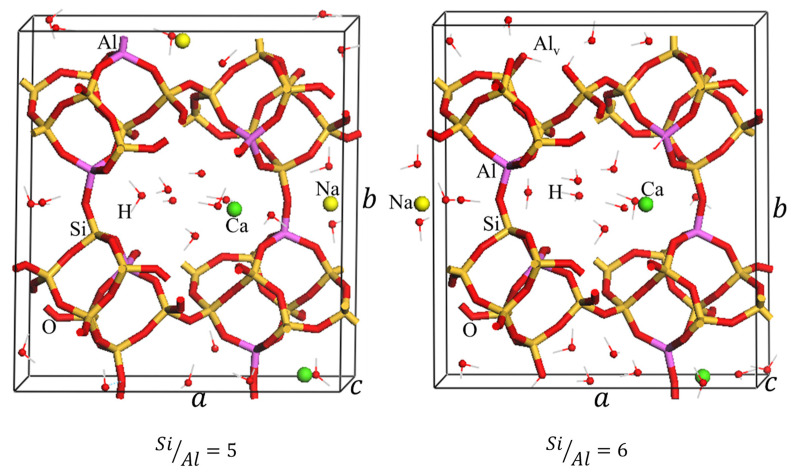

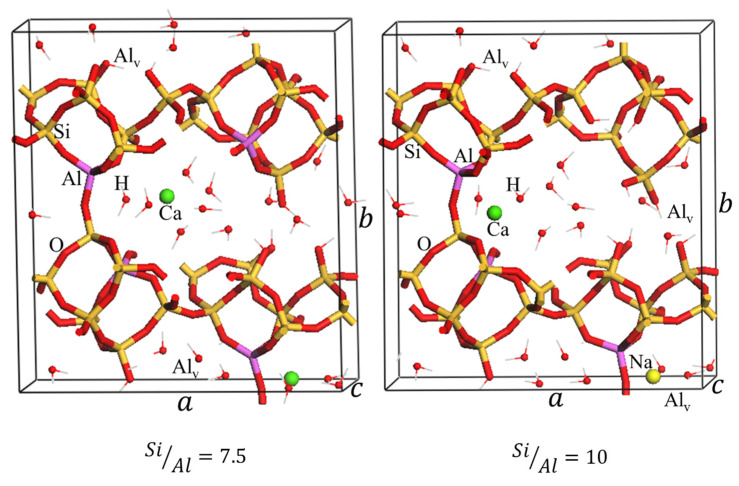
Simulated annealing optimized structure of CLI with Si/Al = 5, 6, 7.5, and 10. Al_v_ represents the aluminum vacancies.

First, we have optimized the structures of CLI taking the ratio Si/Al = 5, 6, 7.5, and 10 by using the simulating annealing method we introduced in a previous work [69]. For this purpose, we have considered the general chemical formula of Clinoptilolite as ${\mathrm{Na}}_{x}{\mathrm{Ca}}_{y}H_{4\left(6-n\right)}{\mathrm{Al}}_{n}{\mathrm{Si}}_{30}O_{72}.24H_{2}O$, where n=x+2y, and n=6;5;4 and 3. Here, the ratio is computed by SiAl=30n. A schematic view of the optimized structures is shown in Figure 8, while the detailed crystal structures can be accessed in cif format in the Supplementary Information (SI).

For Si/Al = 5 of Nat-CLI, we have considered the structure reported in previous works where the authors suggested sodium and calcium unit cells with six aluminum located in T2 and T3 sites [70], and 24 water molecules [69]. For structures with Si/Al = 6, 7.5, and 10, we have considered that the decrease of Al atoms by acid treatment creates vacancies in the framework and also a removal of an extraframework charge. The oxygen atoms next to the vacancy are capped by H^+^. Moreover, it was considered that the number of compensated cations (Na^+^ and Ca^2+^) decreased with the increase of Si/Al ratios as indicated in Figure 1. For instance, if one Al^3+^ ion is removed (the case when Si/Al = 6), four hydrogen ions need to be included in the structure, and a positive charge is also removed, in this case, the sodium ion (Na^+^). In another way, if two Al ions are removed (the case when Si/Al = 7.5), they are replaced by 8H^+^, and 2 positive charges are also removed, which could be 2 Na^+^ ions or one Ca^2+^ ion. In the case that 3 Al^3+^ ions are removed (Si/Al = 10), 12H^+^ must be included in the cell to close the valence of the oxygens and remove 3 positive charges, which could be 3 Na^+^ ions or one Na^+^ ion plus one Ca^2+^ ion.

All configurations were studied and the most stable are shown in Figure 8 with the following formula:

$${\mathrm{Na}}_{2}{\mathrm{Ca}}_{2}{\mathrm{Al}}_{6}{\mathrm{Si}}_{30}O_{72}.24H_{2}O\;\;\frac{\mathrm{Si}}{\mathrm{Al}}=5$$

$$\mathrm{Na}{\mathrm{Ca}}_{2}H_{4}{\mathrm{Al}}_{5}{\mathrm{Si}}_{30}O_{72}.24H_{2}O\;\;\frac{\mathrm{Si}}{\mathrm{Al}}=6$$

$${\mathrm{Na}}_{2}{\mathrm{Ca}H_{8}\mathrm{Al}}_{4}{\mathrm{Si}}_{30}O_{72}.24H_{2}O\;\;\frac{\mathrm{Si}}{\mathrm{Al}}=7.5$$

$$\mathrm{Na}\mathrm{Ca}H_{12}{\mathrm{Al}}_{3}{\mathrm{Si}}_{30}O_{72}.24H_{2}O\;\;\frac{\mathrm{Si}}{\mathrm{Al}}=10$$

It is important to mention that the values of Si/Al used in this study are similar to that obtained from experimental data.

The optimized structural parameters of Clinoptilolite, with Si/Al values from 5 to 10 are shown in Table 6. It can be seen that the cell volume of clinoptilolite slightly increased with increasing the Si/Al ratio from 5 to 7.5, and after that staying almost constant by less than 0.2% up to Si/Al = 10. This result can be attributed to the decrease in the number of Al and the exchanged cations in the structure framework thus reducing the electrostatic attraction between extra-framework cations and the oxygen atoms in the framework [71]. Similar results were reported in another study [72]. In addition, we have to note that we are replacing each Al^3+^ by 4 H^+^, very close to the Al^3+^ vacancy site. So, this creates a very strong electrostatic repulsion which is stabilized by increasing the distance between H^+^ ions and therefore increasing the cell volume. Nevertheless, when Si/Al = 10, this effect is negligible in comparison to Si/Al = 7.5, given that the volume has already increased sufficiently just prior to that point. Overall, the volume of acid-treated clinoptilolite without metallic cations inside of their framework increases with decreasing of Al content [42].

#### 2.2.2. DFT Study of the Sorption of Cd^2+^ and Pb^2+^ by Natural and Dealuminated Clinoptilolites

In this stage, we have studied the sorption of Pb^2+^ and Cd^2+^ in the natural and dealuminated clinoptilolite models obtained above. For this purpose, we have used the simulated annealing protocol for optimizing the structure of clinoptilolite with Si/Al = 5, 6, 7.5, and 10 with cadmium or lead atoms within the structures. The general reactions of the ion exchange process can be expressed by the following general equation:

$${\mathrm{Na}}_{x}{\mathrm{Ca}}_{y}{H_{4(6-n)}\mathrm{Al}}_{n}{\mathrm{Si}}_{30}O_{72}.24H_{2}O+zM^{2+}{{(H}_{2}O)}_{6}+kH_{2}O\to {M_{z}\mathrm{Na}}_{x}{\mathrm{Ca}}_{y}{H_{4(6-n)}\mathrm{Al}}_{n}{\mathrm{Si}}_{30}O_{72}.24H_{2}O+\;(x\;-\;x\prime ){\mathrm{Na}}^{+}{{(H}_{2}O)}_{6}+(y\;-\;y\prime ){\mathrm{Ca}}^{2+}{{(H}_{2}O)}_{8}$$

where, *M* = *Pb* or *Cd*. Here is fulfilled:

0< n ≤6

0 ≤ x′ ≤ x

0 ≤ y′ ≤ y

as well as,

z =2(x − x′)+(y − y′)

n = x +2y

k =2(y − y′) − 6(x − x′)

The exchange energy, per metal atom, of the adsorption process is obtained by $E_{exc}$ follow:

(1) $$E_{\mathrm{exc}}=\frac{1}{z}Q\;Q=E_{M{\mathrm{Na}}_{x\prime }{\mathrm{Ca}}_{y\prime }H_{4\left(6-n\right)}{\mathrm{Al}}_{n}{\mathrm{Si}}_{30}O_{72}.24H_{2}O}+\left(x\;-\;x\prime \right)E_{{\mathrm{Na}}^{+}{\left(H_{2}O\right)}_{6}}+\left(y\;-\;y\prime \right)+\left(y\;-\;y\prime \right)E_{{\mathrm{Ca}}^{2+}{\left(H_{2}O\right)}_{8}}\;-\;E_{{\mathrm{Na}}_{x}{\mathrm{Ca}}_{y}H_{4\left(6-n\right)}{\mathrm{Al}}_{n}{\mathrm{Si}}_{30}O_{72}.24H_{2}O}\;-\;zE_{M^{2+}{\left(H_{2}O\right)}_{6}}$$

Here, $E_{M_{z}{\mathrm{Na}}_{x\prime }{\mathrm{Ca}}_{y\prime }H_{4\left(6-n\right)}{\mathrm{Al}}_{n}{\mathrm{Si}}_{30}O_{72}.24H_{2}O}$ and $E_{{\mathrm{Na}}_{x}{\mathrm{Ca}}_{y}H_{4\left(6-n\right)}{\mathrm{Al}}_{n}{\mathrm{Si}}_{30}O_{72}.24H_{2}O}$ are the energies of the system represented by the cell model after and before the M (Pb or Cd) exchange, respectively. On the other hand, $E_{{\mathrm{Na}}^{+}{\left(H_{2}O\right)}_{6}}$, $E_{{\mathrm{Ca}}^{2+}{\left(H_{2}O\right)}_{8}}$ and $E_{M^{2+}{\left(H_{2}O\right)}_{6}}$ are the solvation energies calculated for cations in a cell model of $20\times 20\times 20\;{Å}^{3}$.

**Figure 9:**
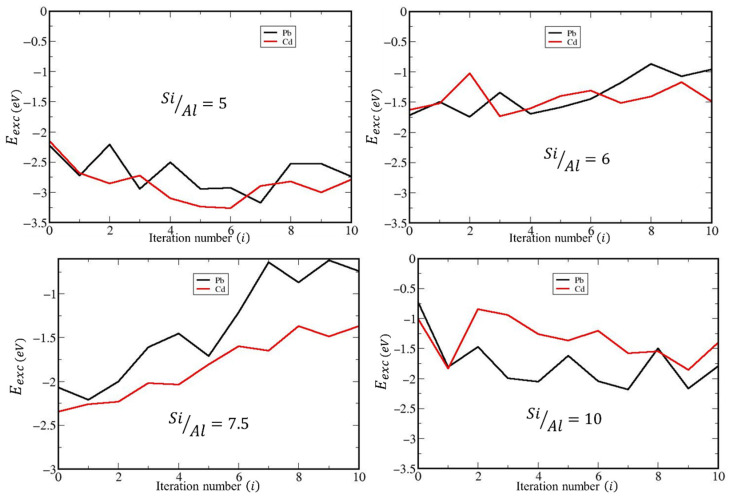
Simulated annealing performance for SiAl=5;6;7.5 and 10. We have represented $E_{exc}$ vs. simulated annealing iterations.

Table 7, as well as Figure 9 shows the most favorable reaction energies of the cation exchange process calculated by DFT as a function of the Si/Al ratio (Si/Al = 5, 6, 7.5, and 10). Overall, when E_exc_ < 0, the adsorption is favorable and the reaction is exothermic, whereas a positive value indicates unfavorable adsorption. It is observed that the exchange energies of natural and dealuminated clinoptilolite are favorable for all iterations for each considered Si/Al ratios. In addition, their absolute values for dealuminated zeolites are reduced with respect to the natural zeolite SiAl=5. These results could be explained by using a structural point of view. It is clear from Table 6, that the variation of volume value is directly connected to the variation of *b* parameter of the cell. In the case of the clinoptilolite, it looks like the distance between layers across (010) crystallographic direction would be the main factor of the volume variation values. So, the increment of *b* would take a direct reduction in the interaction of exchanged metals with the zeolite framework.

**Figure 10:**
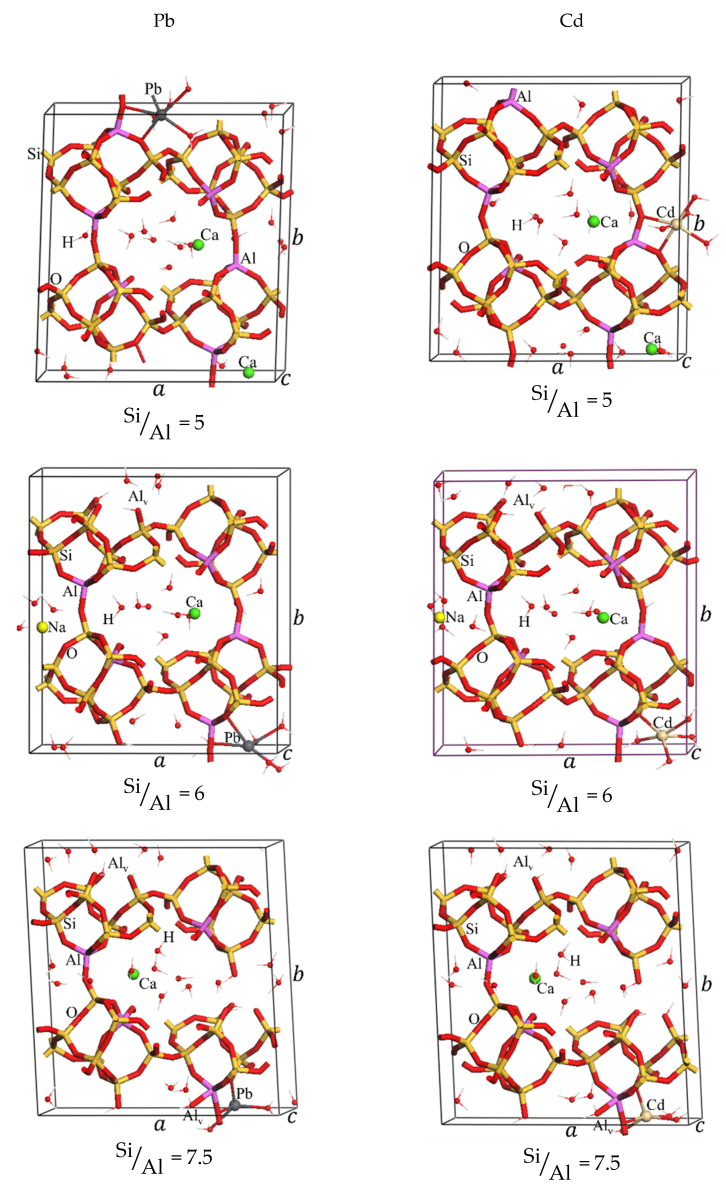

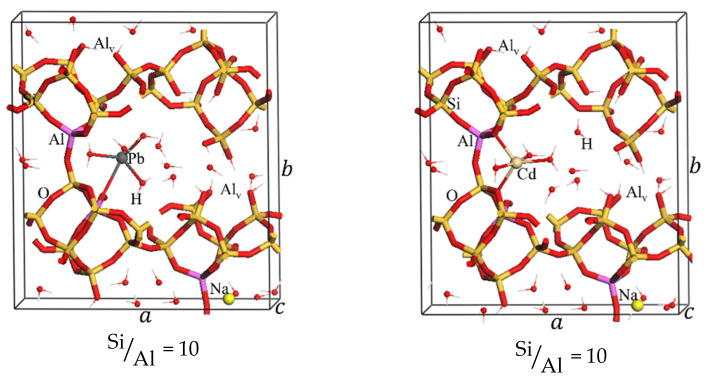
Optimized structure of natural and acidified Clinoptilolite after the adsorption of cadmium and lead.

A view of the simulated annealing curves (Figure 9) indicate the need for such approach for modeling the complex system of a small/medium pore zeolite with water and extra-framework cations. In most cases, the lowest energy iteration, taken here as ${\;E}_{exc}$, does not correspond with that of the optimized initial structures (iteration 0 in Figure 7). The structures of the most stable configurations are displayed in Figure 10, and the cif format is supplied in the Supplementary Material. Large local deformations are expected to occur due to the dealumination processes, as can be seen in Figure 10. The overall shape and structure do not suffer large variations as a consequence of the complex hydrogen bond network connecting both aluminum-vacancy caped hydroxyl groups and water molecules with the framework oxygen atoms (Figure S1 in SI).

The differences in the computed ${\;E}_{\mathrm{exc}}$ (Table 7) do not justify the experimentally observed large preference in all Si/Al studied for capturing Pb as compared to Cd. At this point, it is important to consider that our calculation considers the energy differences of the optimized initial and final systems, thus only providing information on the thermodynamics branch of the reaction. Above, when analyzing the experimental data of metal removal and compared with reported data on fully exchanged Pb and Cd HEU zeolites, we mentioned that kinetics factors could have a relevant role in the experimental selectivity. To consider the kinetics of the reaction, it would require a very heavy calculation for modeling the hydrated cation diffusion through the zeolite, which would be very difficult at the high DFT level used for the above modeling results. Nevertheless, the crystal chemistry of the optimized Pb and Cd exchange structures offers a clue in this respect. From Figure 8 we observe that the Pb and Cd, both, are absorbed inside the zeolite pores in the form $M^{2+}{\left(H_{2}O\right)}_{4}$ of another two coordinate to framework oxygen atoms. So, we have considered the primary step needed for Pb and Cd incorporation into the zeolite from the solution, where both cations are hexa-coordinated. Thus, we have computed the energy needed for the transformation in the sequence

$M^{2+}{\left(H_{2}O\right)}_{6}\to M^{2+}{\left(H_{2}O\right)}_{5}\to M^{2+}{\left(H_{2}O\right)}_{4}$, i.e.,

$$M^{2+}{\left(H_{2}O\right)}_{6}\to M^{2+}{\left(H_{2}O\right)}_{5}+H_{2}O$$

$$M^{2+}{\left(H_{2}O\right)}_{5}\to M^{2+}{\left(H_{2}O\right)}_{4}+H_{2}O$$

So, the partial desolvation energies of hydrated $M^{2+}$ changing from 6→5→4 water molecules are obtained by $E_{reac}^{6\to 5}$ and $E_{reac}^{5\to 4}$, respectively; follow:

(2) $$E_{\mathrm{reac}}^{6\to 5}=E_{M^{2+}{\left(H_{2}O\right)}_{5}}+E_{H_{2}O}\;-\;E_{M^{2+}{\left(H_{2}O\right)}_{6}}\;E_{\mathrm{reac}}^{5\to 4}=E_{M^{2+}{\left(H_{2}O\right)}_{4}}+E_{H_{2}O}\;-\;E_{M^{2+}{\left(H_{2}O\right)}_{5}}$$

The values of $E_{reac}^{6\to 5}$ and $E_{reac}^{5\to 4}$ are shown in Table 8. It can be observed that for the first and second desolvation reactions, the energies of Pb ions are less than for Cd ions. Such results are in agreement with the experimental evidences where high selectivity for Pb over Cd ions takes place. As well as, these results would explain why the Pb get inside the better than Cd in the case of CLI zeolite, i.e., since it is easier to remove two water molecules in Pb than in Cd, so it would be easier to absorb ${Pb}^{2+}{\left(H_{2}O\right)}_{4}$ than ${Cd}^{2+}{\left(H_{2}O\right)}_{4}$. The overall analysis then suggests that the selectivity is kinetically controlled rather than thermodynamics.

**Figure 11:**
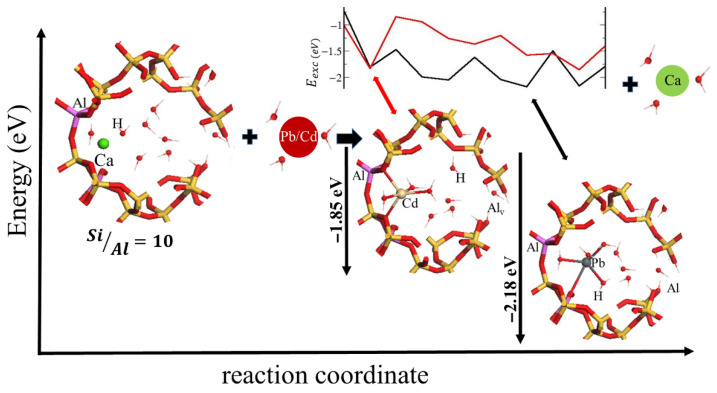
Summarized concept diagram of the ion exchange reaction by using SiAl=10 as an example.

The schematic diagram of the reaction for SiAl=10 ratio is shown in Figure 11 for both, Pb and Cd, ion exchange reactions (Table 7). It is highlighted that for Cd and Pb, the SA cycles number 2 and 7 are those used for computing the exchange reaction energies, respectively. For SiAl=5, 6, 7.5 the ion exchange energies are more favorable for Cd than for Pb. Only for the ratio SiAl=10, it goes in opposite way.

## 3. Materials and Methods

### 3.1. Experiments

Stock solutions of Pb^2+^ and Cd^2+^ (1000 mg/L) were prepared by dissolving 0.337 g of PbCl_2_ (99%, Sigma Aldrich, St. Louis, MO, USA) and 0.508 g of CdCl_2_·2.5H_2_O (79.9%, Sigma Aldrich) in deionized water, respectively. By diluting the stock solutions with deionized water, solutions of 25 to 500 mg/L were prepared. Pb^2+^ and Cd^2+^ calibration curves were obtained by dilution of 1000 mg/L standard solutions of the metals in 2% *v*/*v* HNO_3_ (Fluka Analytical, Buchs, Switzerland).

Dealumination of CLI was performed by washing the samples using H_2_SO_4_ (Fermont, CAS 7664.93.9) solutions at 0.1 M, 0.2 M, 0.5 M, and 1.0 M for 2 h at 90 °C under reflux condition, with a ratio of 1:20 W/V. Afterward, the samples were washed for several times with deionized water (resistivity, 18.2 MΩ cm) until the pH of washing solution reached approximately 6. Finally, the samples were dried in a furnace (Felisa, model 291) at 80 °C for 12 h, and named as CLI-0.1 M, CLI-0.2 M, CLI-0.5 M, and CLI-1.0 M.

Characterization of the natural and dealuminated samples was performed using an APD 2000 PRO X-ray diffractometer with CuKα radiation. The patterns were recorded between 6° and 60° 2θ with a scanning speed of 0.025 deg s^−1^ and a step time of 10 s. 

The morphology of CLI and dealuminated samples were characterized at 20 kV by a JEOL JSM-IT300 scanning electron microscope (SEM), equipped with an energy-dispersive X-ray spectroscopy (EDS) used for analyzing the chemical composition.

Kinetic studies of Pb^2+^ and Cd^2+^ were performed using the batch technique at 25 °C. 10 mL of aqueous solutions of Pb^2+^ and Cd^2+^ at initial concentrations of 100 mg/L were separately contacted with 0.1 g of natural and dealuminated zeolites and stirred at 150 rpm using a rotating incubator. The contact time was varied from 5 to 1440 min to study the effect of contact time on the sorption capacity of Pb^2+^ and Cd^2+^. Metal concentrations in the supernatants were analyzed by flame atomic absorption spectrometry (Thermos Scientific iCE 3000 series, Waltham, MA, USA) after each contact time. The supernatants were collected by centrifugation at 4500 rpm for 2 min. The amount of Pb^2+^ and Cd^2+^ sorbed by CLI and acid-treated samples (meq/g) was calculated using the mass balance expression (3):

(3) $$q=\frac{\left(C_{0}\;-\;C_{f}\right)V}{W}$$

where: *V* is the solution volume (L), *W* is the amount of sorbent (g), C_0_ and C_f_ are the initial and final metal concentration, meq/L.

Equilibrium sorption of Pb^2+^ and Cd^2+^ were done at 25 °C with 0.1 g of natural and acid-treated zeolites in contact in a conical flask with 10 mL of Pb^2+^ or Cd^2+^ at initial concentrations ranging from 10 to 500 mg/L and at pH approximately 6. The mixtures were agitated in an orbital shaker at 150 rpm for 12 h. Then, the samples were centrifuged at 4500 rpm for 2 min and the equilibrium concentrations of the remaining Pb^2+^ and Cd^2+^ were analyzed by Flame Atomic Absorption Spectrometry. All experiments were performed in duplicate and the results were expressed as averaged values. The present study examined the equilibrium and kinetic behavior of lead and cadmium ions in a single-component system.

The kinetics data obtained were evaluated using nonlinear equations of pseudo-first-order (PFO) and pseudo-second-order (PSO) models (Equations (4) and (5)) applying the solver add-in from Microsoft’s spreadsheet tool of Microsoft Excel.

(4) $$q_{t}=q_{e,\;\mathrm{cal}}\left(1\;-\;\exp(-k_{1}t\right))$$

(5) $$q_{t}=\frac{k_{2}{q_{e,\mathrm{cal}}}^{2}t}{1+k_{2}q_{e,\mathrm{cal}}t}$$

where q_e_,_cal_ is the theoretical adsorption capacity (meq/g), k_1_ (1/min), and k_2_ (g/(meq.min)) are the pseudo-first and pseudo-second order rate constants, respectively.

The equilibrium data obtained were adjusted to Langmuir and Freundlich nonlinear models using the solver add-in from Microsoft’s spreadsheet tool of Microsoft Excel. The Equations (6) and (7) correspond to the Langmuir and Freundlich nonlinear models, respectively.

(6) $$q_{e}=\frac{q_{m}K_{L}C_{e}}{1+K_{L}C_{e}}$$

(7) $$q_{e}=K_{F}{C_{e}}^{\frac{1}{n}}$$

where K_L_ (L/meq) is the Langmuir equilibrium constant, and q_m_ (meq/g) is the maximum adsorption capacity. *n* (dimensionless) and K_F_ (mg/g)(L/mg)^1/n^ are the exponent and the Freundlich parameter, respectively.

To confirm a good fit with the proposed kinetic and isotherm models, four error functions, such as mean percentage error (ARE), nonlinear chi-square test (*χ*^2^), residual root mean square error (RMSE), normalized standard deviation (Δ*q*(%) [72] were calculated with the Equations (8)–(11) given below:

(8) $$\mathrm{ARE}\;\left(\%\right)=\frac{100}{N}\sum_{i=1}^{N}\left|\frac{q_{e,\exp}\;-\;q_{e,\mathrm{calc}}}{q_{e,\exp}}\right|$$

(9) $$\chi^{2}=\sum_{i=1}^{N}\frac{{\left(q_{e,\exp}\;-\;q_{e,\mathrm{calc}}\right)}^{2}}{q_{e,\mathrm{calc}}}$$

(10) $$\mathrm{RMSE}=\sqrt{\frac{1}{N\;-\;2}\sum_{i=1}^{N}{\left(q_{e,\exp}\;-\;q_{e,\mathrm{calc}}\right)}^{2}}$$

(11) $$\Delta q\left(\%\right)=100\sqrt{\frac{1}{N\;-\;1}\sum_{i=1}^{N}({\frac{q_{e,\exp}\;-\;q_{e,\mathrm{calc}}}{q_{e,\exp}})}^{2}}$$

where *q*_e,exp_ is the adsorption capacity obtained from the batch experiment (meq/g), *q*_e,calc_ is the adsorption capacity obtained with a mathematical model corresponding (meq/g), and *N* is the corresponding number of observations in the experiment. Lower values of ARE, *χ*^2^, RMSE, and Δ*q*(%) indicate the best fit of the model.

### 3.2. DFT-Based Simulated Annealing Method

DFT periodic calculations were performed in order to study the ion exchange process of hydrated CLI with different Si/Al ratios. The calculations were done in a periodic framework, as implemented in the Vienna Ab-initio Simulation Package (VASP) [73,74]. The projector augmented wave (PAW) method [75,76,77] was used to describe the frozen core electrons and their interaction with the valence electrons. A Γ-centred grid of k-points was used for integrations in reciprocal space, where the smallest allowed spacing between k-points was set at 0.5 Å^−1^ in the Brillouin zone corresponding to the primitive cell. In order to describe the electron interactions, we have used the generalized gradient approximation of the Perdew–Burke–Ernzerhof parametrization form (GGA-PBE), [77] and corrected for dispersion interactions (DFT-D3) by Grimme et al. [78]. The electronic wave function was expanded in plane waves up to a cut-off energy of 500 eV. The annealing process implemented in our work consists of n iterations, as described elsewhere.1 Each one goes in the form: ramp up + ramp down + geometry optimization. The ramp-up employs a Molecular Dynamic (MD) simulation at a constant number of particles and constant volume while controlling the temperature by rescaling velocities of all particles according to the temperature in the range 1K→T In all cases, energy relaxation was minimized until a change in the total (free) energy was smaller than 0.001 eV.

## 4. Conclusions

This work shows experimental and theoretical results in order to investigate the exchange of heavy metals Cd^2+^ and Pb^2+^ in hydrated-clinoptilolite for different Si/Al ratios. We obtained here that the amount of exchangeable cations and sorption capacity of Pb^2+^ and Cd^2+^ decrease with increasing of Si/Al ratio. In this way, it is not explained from a thermodynamic point of view since very close values for the energy of the ion exchange reactions were theoretically obtained. However, it could be understood from a kinetic perspective. We concluded that the pores architectures of the zeolite modify the first hydration coordination sphere of heavy metals from six in the bulk water to four in the zeolite framework and two oxygen atoms from the zeolite. It looks like the involved energy in the loss of these two water molecules is essential for the large selectivity in favor of Pb^2+^. In this way, this shows the importance of the kinetic factor in order to understand the sorption mechanism. From a methodological point of view, we have shown that the uses of (DFT)-based simulated annealing (SA) [69] take special importance in the case of ionic exchange of heavy metal by zeolites. With respect to prospective metal removal applications, our results suggest that further modifications would be required to selectively trap cadmium relative to lead. Work is underway to design new modifications for this purpose.

## Figures and Tables

**Table 1:** EDS analysis of natural and acid-treated clinoptilolites.

Weight % of Elements	Zeolites				
	Nat-CLI	CLI-0.1 M	CLI-0.2 M	CLI-0.5 M	CLI-1.0 M
Na	0.56	0.0	0.0	0.0	0.0
K	1.17	0.97	0.58	0.51	0.46
Ca	1.65	0.89	0.68	0.50	0.22
Mg	0.21	0.15	0.0	0.0	0.0
Fe	0.37	0.23	0.21	0.11	0.07
Si	25.77	26.44	27.78	28.85	27.71
Al	5.90	4.98	3.51	3.19	2.91
O	64.37	66.34	67.24	66.84	68.63
Si/Al	4.37	5.31	7.91	9.04	9.52

**Table 2:** PFO and PSO kinetic parameters of Pb^2+^ and Cd^2+^ adsorption by untreated and dealuminated zeolites.

Non-Linear Pseudo First Order Model								
Zeolite	q_e,exp_ (×10^−3^) (meq/g)		q_e,cal_ (×10^−3^) (meq/g)		k_1_ (1/min)		R^2^	
	Pb^2+^	Cd^2+^	Pb^2+^	Cd^2+^	Pb^2+^	Cd^2+^	Pb^2+^	Cd^2+^
Nat-CLI	49.118	19.536	48.168	18.915	0.084	0.120	0.899	0.686
CLI-0.1 M	47.181	18.015	44.995	16.272	0.038	0.021	0.890	0.822
CLI-0.2 M	46.103	14.154	43.231	13.693	0.030	0.009	0.828	0.938
CLI-0.5 M	29.140	11.590	27.396	10.735	0.018	0.042	0.916	0.662
CLI-1.0 M	20.309	12.623	18.317	11.097	0.011	0.019	0.806	0.775
Non-linear pseudo second order model								
	q_e,exp_ (×10^−3^) (meq/g)		q_e,cal_ (×10^−3^) (meq/g)		k_2_ (g/(meq min))		R^2^	
	Pb^2+^	Cd^2+^	Pb^2+^	Cd^2+^	Pb^2+^	Cd^2+^	Pb^2+^	Cd^2+^
Nat-CLI	49.118	19.536	49.663	19.296	3.614	17.144	0.993	0.976
CLI-0.1 M	47.181	18.015	47.846	17.772	1.261	1.702	0.983	0.952
CLI-0.2 M	46.103	14.154	46.607	15.255	0.966	0.914	0.962	0.981
CLI-0.5 M	29.140	11.590	30.189	11.401	0.876	5.907	0.978	0.912
CLI-1.0 M	20.309	12.623	20.303	12.258	0.783	2.184	0.921	0.926

**Table 3:** Isotherm parameters obtained by nonlinear model for Pb^2+^ and Cd^2+^ sorption using natural and dealuminated zeolites.

Nonlinear Langmuir Isotherm Model						
Zeolite	q,_max,_ (×10^−3^) (meq/g)		K_L_ (×10^−2^) (L/meq)		R^2^	
	Pb^2+^	Cd^2+^	Pb^2+^	Cd^2+^	Pb^2+^	Cd^2+^
Nat-CLI	224.554	39.044	8.429	0.125	0.964	0.985
CLI-0.1 M	209.044	28.596	4.666	0.184	0.977	0.988
CLI-0.2 M	107.284	31.995	5.127	0.084	0.979	0.986
CLI-0.5 M	56.541	27.403	0.197	0.092	0.920	0.980
CLI-1.0 M	53.802	20.529	0.103	0.142	0.948	0.990
Nonlinear Freundlich isotherm model						
	n		K_F_ (L/g)		R^2^	
	Pb^2+^	Cd^2+^	Pb^2+^	Cd^2+^	Pb^2+^	Cd^2+^
Nat-CLI	2.653	2.344	35.204	1.008	0.918	0.960
CLI-0.1 M	3.112	2.701	31.578	1.233	0.836	0.914
CLI-0.2 M	3.908	2.059	19.974	0.460	0.953	0.960
CLI-0.5 M	2.150	2.216	1.429	0.524	0.980	0.953
CLI-1.0 M	1.766	2.653	0.515	0.789	0.977	0.941

**Table 4:** Comparation of maximum adsorption capacity of Pb^2+^ by natural and modified zeolites.

Type of Zeolite		Qmax (meq/g)	Experimental Conditions	Reference
Natural zeolite	Kazakhstanil zeolite	0.068	pH = 6 Contact time = 4 h T (°C) = 25 Dose (g/L) = 1	[62]
	Ukraine clinoptilolite	0.067	pH = 6.2 Contact time = 48 h T(°C) = 20 Dose (g/L) = 0.5	[17]
	Clinoptilolite	0.931	pH = 4.5 Contact time = 4 h T (°C) = 22 Dose (g/L) = 0.5	[59]
	Scolecite	0.028	pH = 6.0 Contact time = 24 h T (°C) = 25 Dose (g/L) = 5	[60]
	Mexican clinoptilolite	0.701	pH = 5.0 Contact time = 48 h T (°C) = 30 Dose (g/L) = 0.1	[63]
	Clinoptilolite	0.035	pH = 5.0 Contact time = 8 h T (°C) = 25 Dose (g/L) = 5	[61]
	Clinoptilolite	0.319	pH = 4.24 Contact time = 24 h T (°C) = 60 Dose (g/L) = 20	[58]
	Clinoptilolite	0.224	pH = 5.6 Contact time = 12 h T (°C) = 25 Dose (g/L) = 10	In this study
Chemically modified zeolite	NaCl treated	0.591	pH = 4.5 Contact time = 4 h T (°C) = 22 Dose (g/L) = 20	[59]
	Fe(III)-modified zeolite	0.642	pH = 4.24 Contact time = 24 h T (°C) = 60 Dose (g/L) = 20	[58]
	Magnetically Modified Zeolite	0.406	pH = 4 T (°C) = 20 Dose (g/L) = 2	[64]
	NaOH treated zeolite	0.929	pH = 6 Contact time = 2 h T (°C) = 20 Dose (g/L) = 1	[57]
	Acidified clinoptilolites (Si/Al =4.5 –9.5)	0.054–0.209	pH = 5.6 Contact time = 12 h T (°C) = 25 Dose (g/L) = 10	In this study

**Table 5:** Comparation of maximum adsorption capacity of Cd^2+^ by natural and modified zeolites.

Type of Zeolite		Qmax (meq/g)	Experimental Conditions	Reference
Natural zeolite	Ukraine clinoptilolite	0.020	pH = 6.2 Contact time = 48 h T(°C) = 20 Dose (g/L) = 2.5	[17]
	Scolecite	0.002	pH = 6.0 Contact time = 24 h T (°C) = 25 Dose (g/L) = 5	[60]
	Brazilian scolecite	0.893	pH = 6.0 Contact time = 24 h T (°C) = 25 Dose (g/L) = 16	[65]
	Clinoptilolite	0.041	pH = 6.0 Contact time = 6 h T (°C) = 22 Dose (g/L) = 10	[66]
	Clinoptilolite	0.039	pH = 5.6 Contact time = 12 h T (°C) = 25 Dose (g/L) = 10	In this study
Chemically modified zeolite	Iron-coated zeolite	0.065	pH = 6.5 Contact time = 24 h T (°C) = 25 Dose (g/L) = 1	[67]
	NaCl modified zeolite	0.112	pH = 6.0 Contact time = 48 h T (°C) = 25 Dose (g/L) = 10	[68]
	Surfactant-modified zeolite	0.090	pH = 6.0 Contact time = 48 h T (°C) = 25 Dose (g/L) = 10	[68]
	Acidified clinoptilolites (Si/Al =4.5–9.5)	0.021–0.028	pH = 5.6 Contact time = 12 h T (°C) = 25 Dose (g/L) = 10	In this study

**Table 6:** Unit cell parameters and angles of the natural and acid-treated clinoptilolites according to the SiAl ratio.

SiAl	a(Å)	b(Å)	c(Å)	$V({Å}^{3})$	$\alpha \left({}^{\circ}\right)$	$\beta \left({}^{\circ}\right)$
5	17.545	18.214	7.427	2121.2	89.13	116.60
6	17.512	18.349	7.421	2128.3	90.48	116.69
7.5	17.631	18.514	7.434	2169.4	90.67	116.47
10	17.683	18.577	7.410	2166.6	90.48	117.04

**Table 7:** Energy,${\;E}_{exc}$, of the ion exchange reactions.

SiAl	$x\left(\mathrm{Na}\right)$	$y\left(\mathrm{Ca}\right)$	$x\prime \left(\mathrm{Na}\right)$	$y\prime \left(\mathrm{Ca}\right)$	$\mathrm{Pb}\left(\mathrm{eV}\right)$	$\mathrm{Cd}\left(\mathrm{eV}\right)$
5	2	2	0	2	−3.17	−3.26
6	1	2	1	1	−1.60	−1.64
7.5	0	2	0	1	−2.21	−2.34
10	1	1	1	0	−2.18	−1.85

**Table 8:** Desolvation energies of hydrated $M^{2+}$ changing from 6→5→4 water molecules computed by Equation (2).

Ions	$E_{reac}^{6\to 5}$ (eV)	$E_{reac}^{5\to 4}$ (eV)
Pb^2+^	0.88	1.13
Cd^2+^	1.05	1.25

## Data Availability

For any additional information, please contact the corresponding authors.

## Supplementary Materials

The following supporting information can be downloaded at: https://www.mdpi.com/article/10.3390/ijms26094154/s1.
