# Supplementary material for: Further Insight in the High Selectivity of Pb2+ Removal over Cd2+ in Natural and Dealuminated Rich-Clinoptilolite
Source: Int J Mol Sci. 2025 Apr 27;26(9):4154. doi: 10.3390/ijms26094154 (PMC12071763; doi:10.3390/ijms26094154)
Supplement: Supplementary file 1 [file ijms-26-04154-s001.zip › ijms-3563255-supplementary.pdf]

Supporting Information

Further insight in the high selectivity of Pb^{2+} removal over Cd^{2+} in natural and dealuminated rich-clinoptilolite

Yaneth Stephanie Durán Avendaño[1], Norge Cruz Hernández,[2*] A. Rabdel Ruiz-Salvador [3] and Mohamed Abatal[1]**

[1] Facultad de Ingeniería, Universidad Autónoma del Carmen, C.P. 24155, Ciudad del Carmen, Campeche, México.; 090288@mail.unacar.mx.

[2] Departamento de Física Aplicada I, Escuela Politécnica Superior, Universidad de Sevilla, Seville, E-41011, Spain. norge@us.es

[3] Departamento de Sistemas Físicos, Químicos y Naturales, Universidad Pablo de Olavide, Carretera de Utrera km. 1, Seville, E-41013, Spain; Centro de Nanociencia y Tecnologías Sostenibles (CNATS), Universidad Pablo de Olavide, Carretera de Utrera km. 1, Seville, E-41013, Spain

* Correspondence author 1: norge@us.es, (N.C.H)

** Correspondence author 2: mabatal@pampano.unacar.mx, (M.A)

CONTENT :

Table S1. Values of error functions of the kinetic models for Pb^{2+}.

Zeolite	Kinetic models	Error functions				
		ARE	Δ(%)	$\chi 2$	EABS	RMSE
Nat-CLI	PFO	2.223	3.128	0.370	9.867	1.452
	PSO	0.821	1.120	0.051	3.685	0.534
CLI-0.1M	PFO	5.414	9.481	2.777	18.027	2.858
	PSO	2.382	4.035	0.419	7.528	1.218
CLI-0.2M	PFO	8.644	13.733	5.756	26.750	3.898
	PSO	4.196	6.766	1.176	12.897	1.960
CLI-0.5M	PFO	9.079	15.721	3.853	14.151	2.160
	PSO	4.234	6.922	0.646	7.470	1.129
CLI-1.0M	PFO	17.480	25.528	8.389	17.695	2.280
	PSO	10.835	16.688	2.638	11.085	1.511

Table S2. Values of error functions of the kinetic models for Cd^{2+}.

Zeolite	Kinetic models	Error functions				
		ARE	Δ(%)	$\chi2$	EABS	RMSE
Nat-CLI	PFO	2.669	3.258	0.171	4.909	0.631
	PSO	1.471	1.728	0.049	2.726	0.337
CLI-0.1M	PFO	10.611	16.483	3.109	12.169	1.701
	PSO	6.002	9.422	0.827	6.702	0.931
CLI-0.2M	PFO	12.360	21.631	3.210	7.180	1.028
	PSO	6.152	9.972	0.533	4.668	0.599
CLI-0.5M	PFO	9.273	12.816	1.244	8.274	1.119
	PSO	5.308	7.399	0.399	4.722	0.645
CLI-1.0M	PFO	13.619	19.256	2.883	10.247	1.346
	PSO	8.310	12.277	0.962	6.025	0.813

Table S3. Values of error functions of the Langmuir and Freundlich isotherm models for Pb^{2+}.

Zeolite	Kinetic models	Error functions				
		ARE	Δ(%)	$\chi2$	EABS	RMSE
Nat-CLI	Langmuir	16.394	23.987	29.592	83.124	15.777
	Freundlich	76.989	125.118	68.764	182.271	23.777
CLI-0.1M	Langmuir	14.892	23.165	12.440	66.524	11.720
	Freundlich	113.156	193.877	98.572	231.134	31.239
CLI-0.2M	Langmuir	17.702	27.406	12.965	41.286	6.699
	Freundlich	38.584	71.492	18.378	70.913	10.007
CLI-0.5M	Langmuir	26.227	37.652	21.505	32.670	4.663
	Freundlich	7.506	10.444	1.584	13.689	2.351
CLI-1.0M	Langmuir	22.449	30.821	8.242	20.967	2.832
	Freundlich	8.141	11.779	1.485	10.530	1.875

Table S4. Values of error functions of the Langmuir and Freundlich isotherm models for Cd^{2+}.

Zeolite	Kinetic models	Error functions				
		ARE	Δ(%)	$\chi2$	EABS	RMSE
Nat-CLI	Langmuir	6.753	9.708	0.699	8.923	1.391
	Freundlich	25.639	54.114	4.121	14.494	2.232
CLI-0.1M	Langmuir	11.466	18.950	1.076	8.401	1.124
	Freundlich	36.996	68.817	5.880	20.517	2.636
CLI-0.2M	Langmuir	8.132	11.475	0.829	6.447	1.034
	Freundlich	19.975	30.843	2.522	13.493	1.756
CLI-0.5M	Langmuir	12.505	15.263	1.731	13.618	1.883
	Freundlich	21.274	28.715	2.943	16.729	2.252
CLI-1.0M	Langmuir	6.499	8.546	0.308	4.500	0.592
	Freundlich	22.098	44.836	2.438	9.639	1.440

Figure S1. Hydrogen bond network in natural and dealuminated Cd and Pb clinoptilolite.

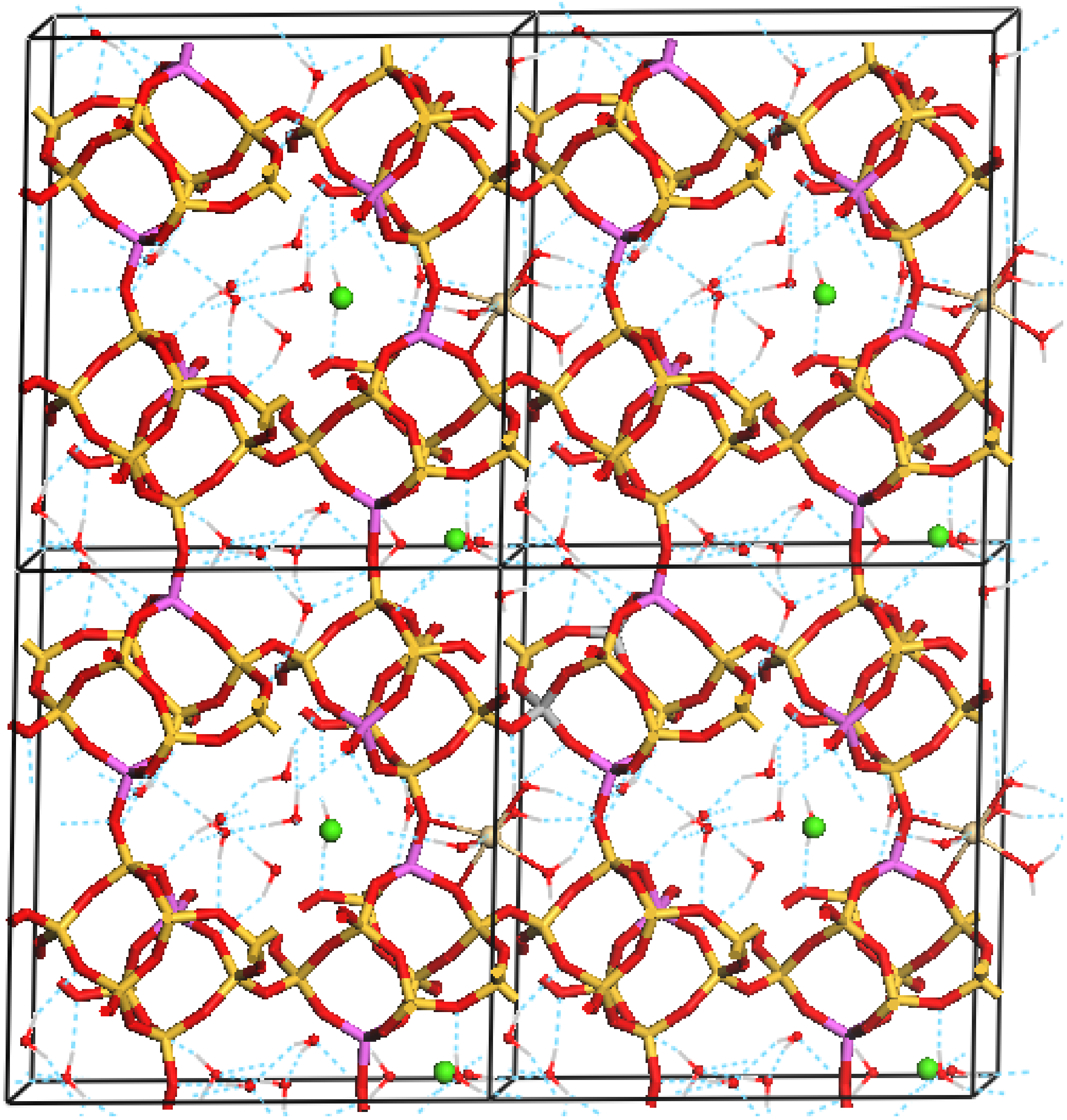

Cd -Clinoptilolite $^{Si}/_{Al} = 5$

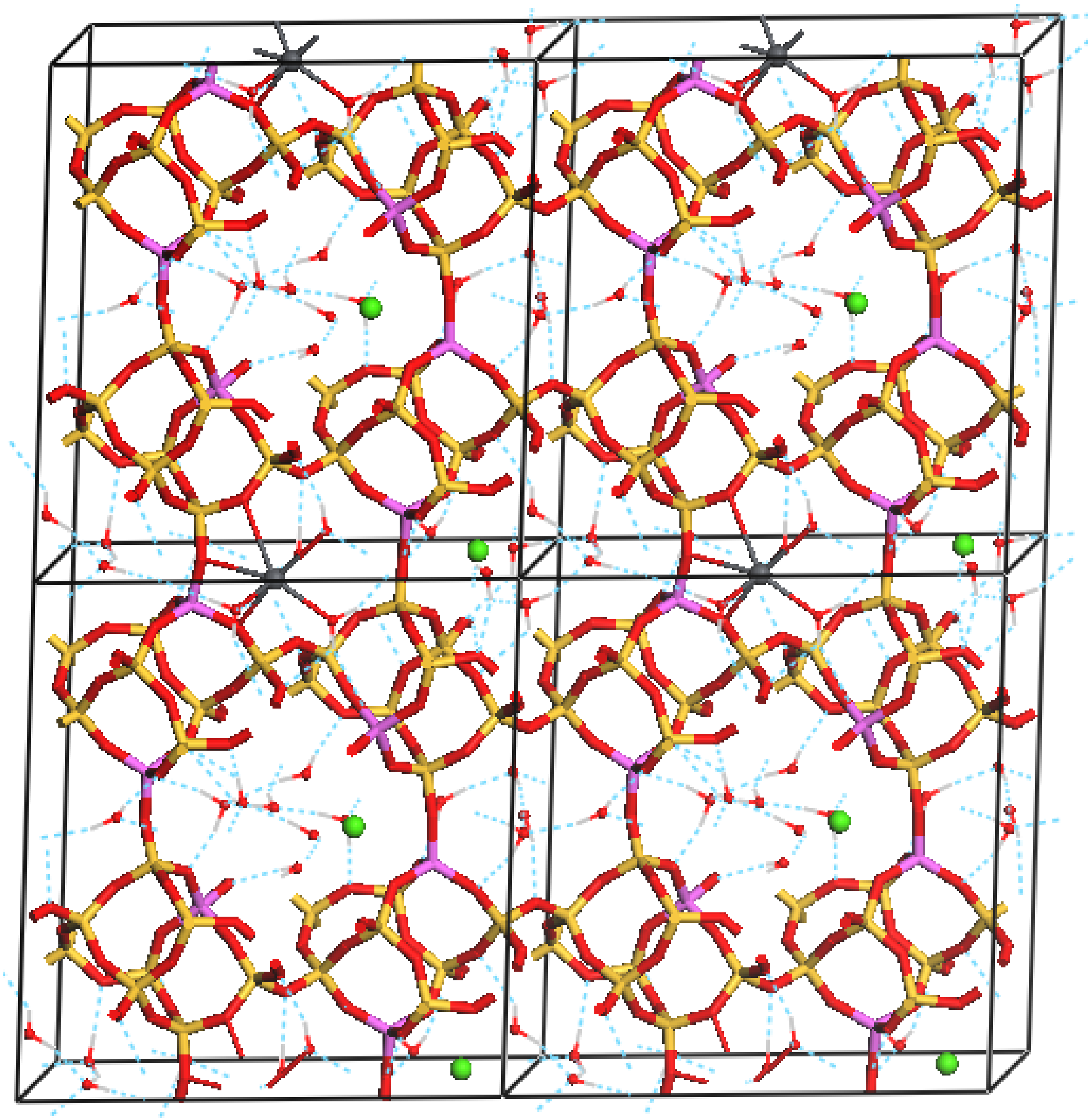

Pb -Clinoptilolite $^{Si}/_{Al} = 5$

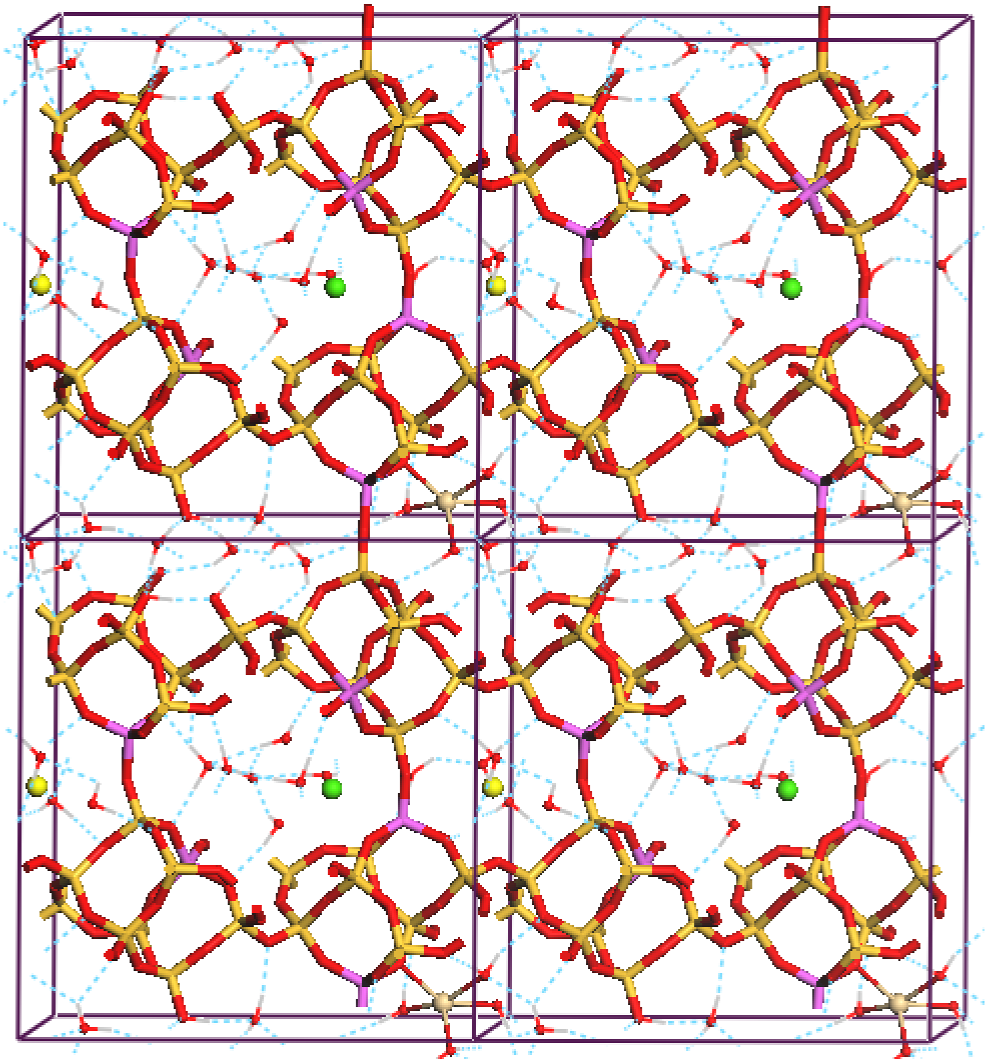

Cd -Clinoptilolite $^{Si}/_{Al} = 6$

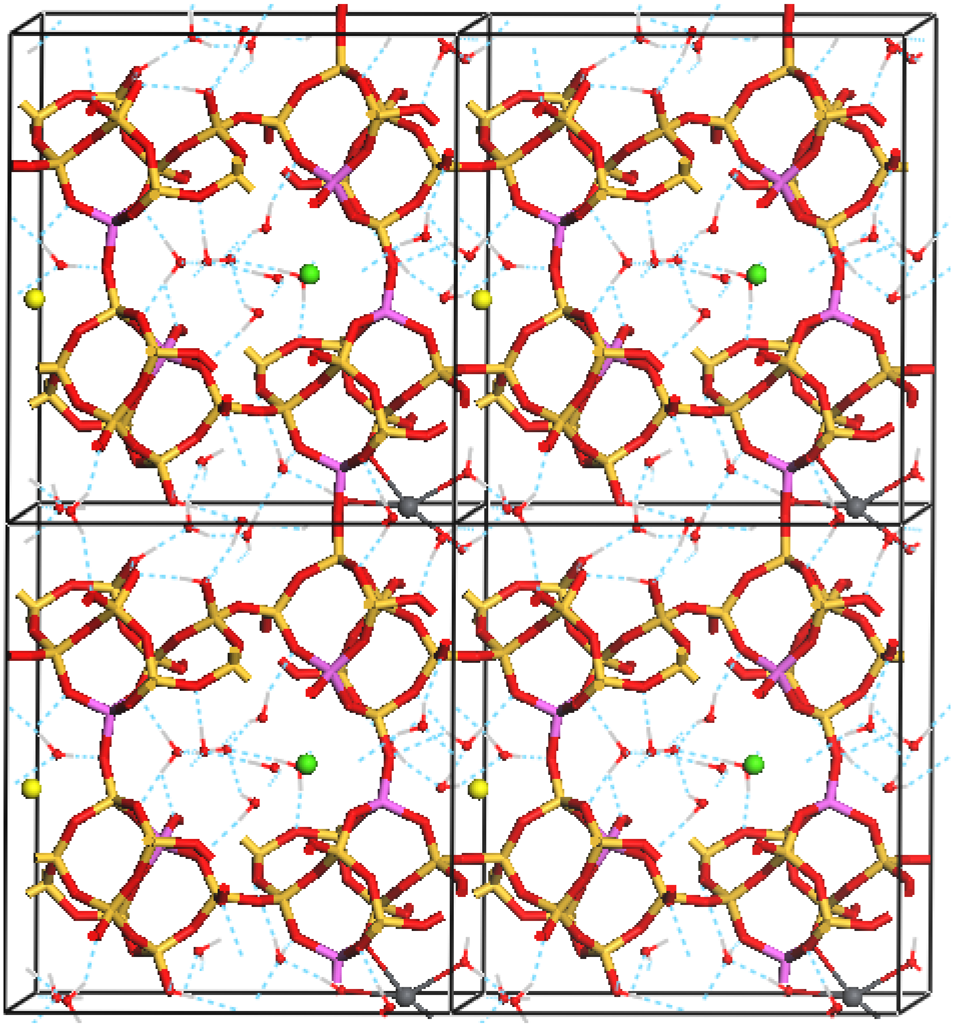

Pb -Clinoptilolite $^{Si}/_{Al} = 6$

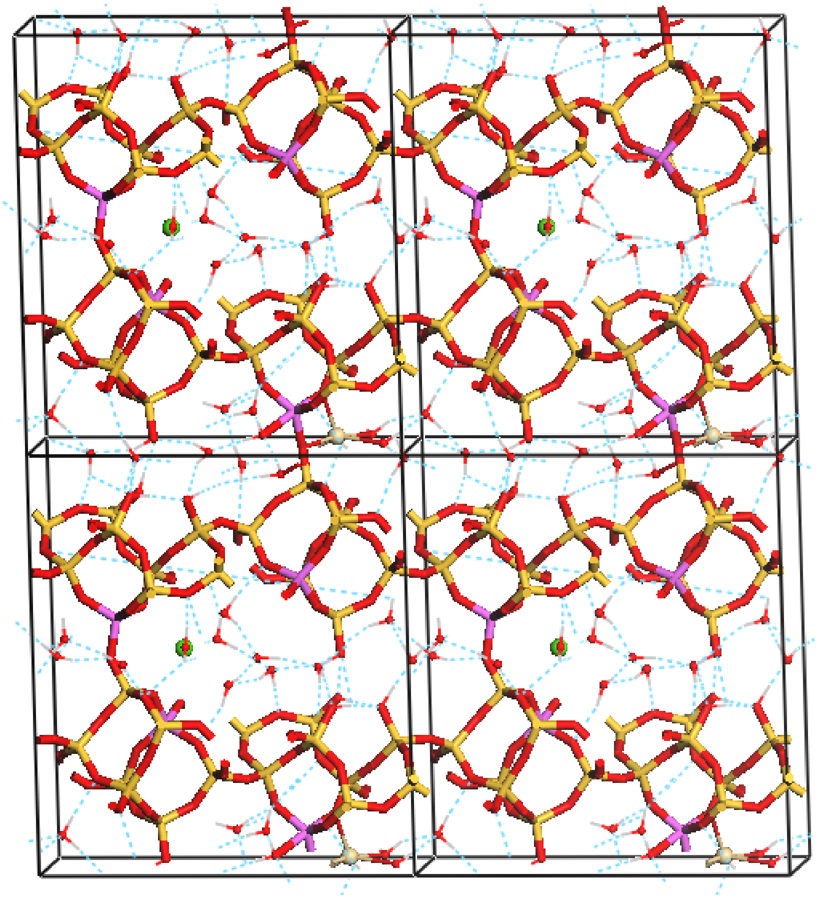

Cd -Clinoptilolite $^{Si}/_{Al} = 7.5$

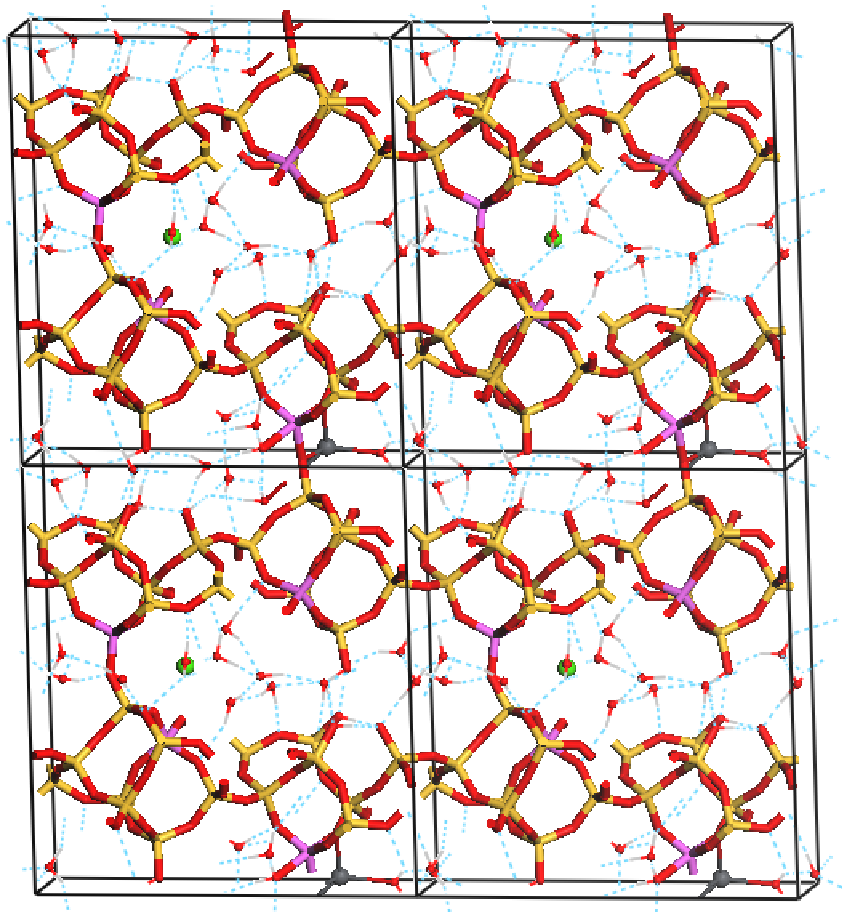

Pb -Clinoptilolite $^{Si}/_{Al} = 7.5$

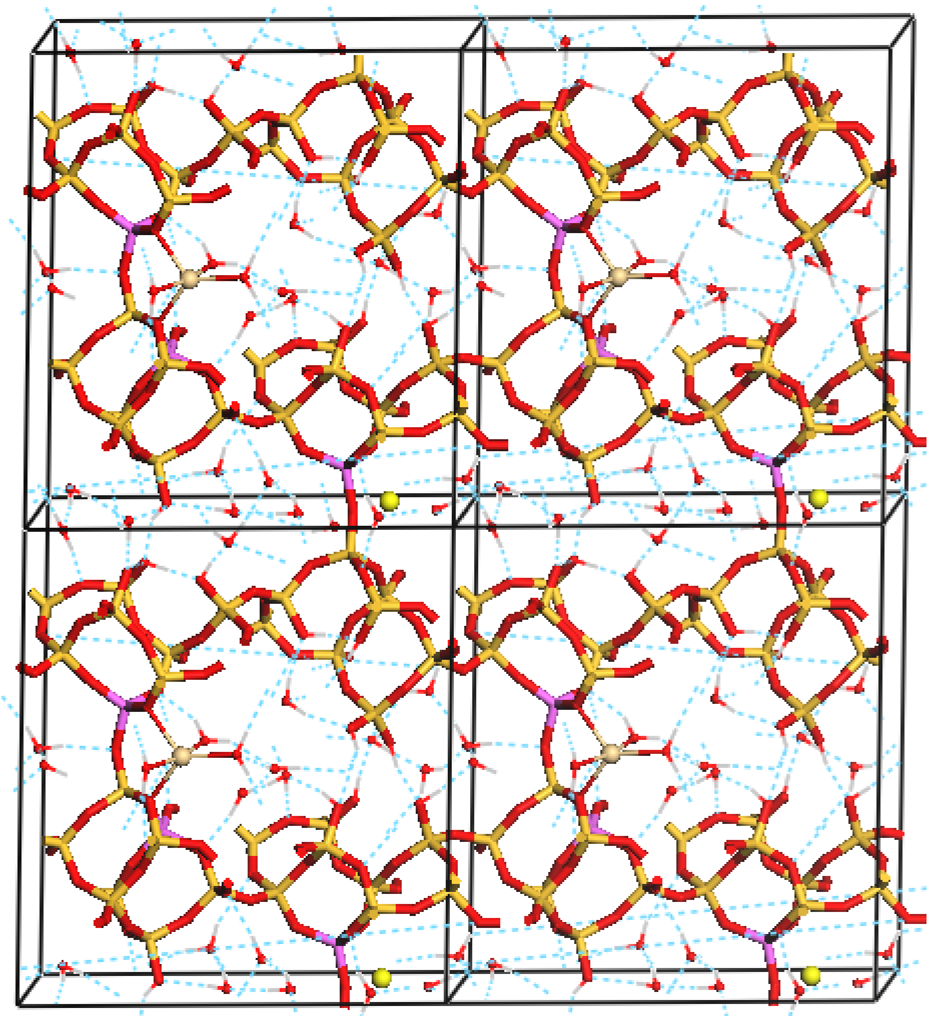

Cd -Clinoptilolite $^{Si}/_{Al} = 10$

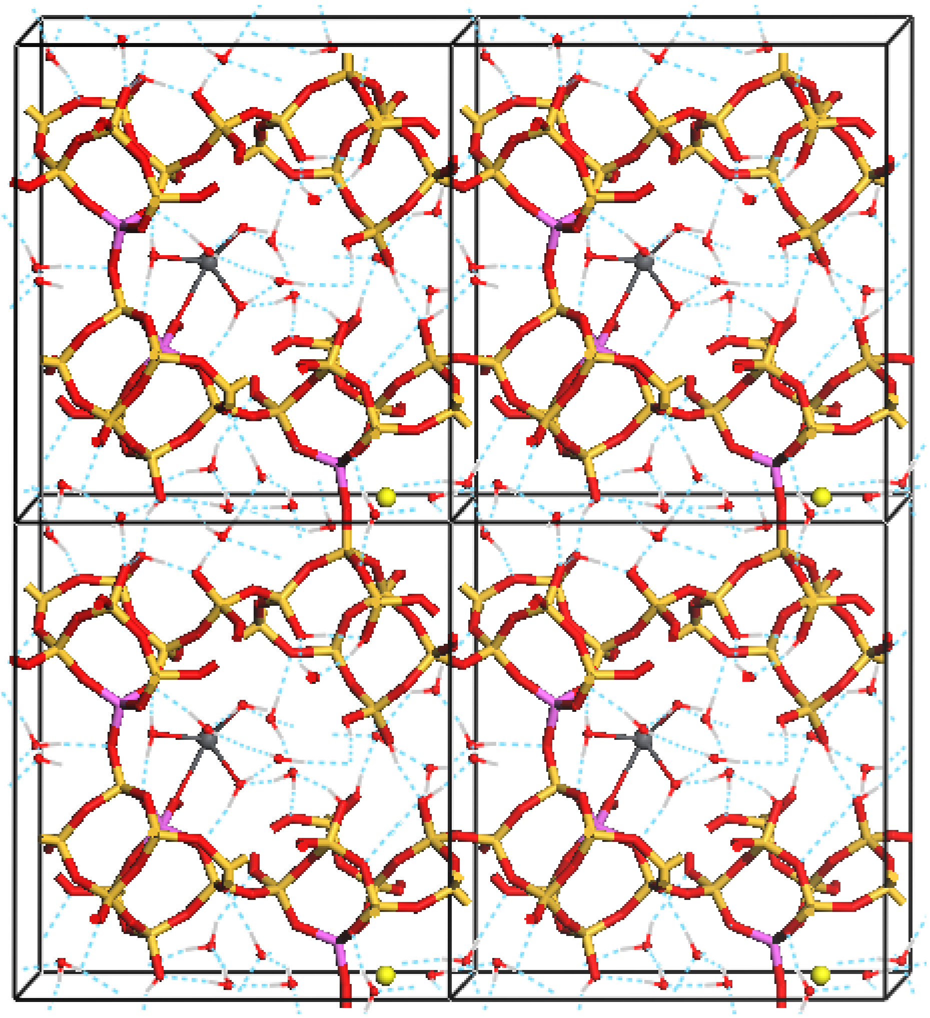

Pb -Clinoptilolite ${}^{Si}/_{Al} = 10$
